# Use of contact precautions for multidrug-resistant organisms and the impact of the COVID-19 pandemic: An Emerging Infections Network (EIN) survey

**DOI:** 10.1017/ash.2023.374

**Published:** 2023-09-29

**Authors:** Jessica Howard-Anderson, Lindsey Gottlieb, Susan E. Beekmann, Philip Polgreen, Jesse T. Jacob, Daniel Z. Uslan

## Abstract

**Background:** The CDC recommends routine use of contact precautions for patients infected or colonized with multidrug-resistant organisms (MDROs). There is variability in implementation of and adherence to this recommendation, which we hypothesized may have been exacerbated by the COVID-19 pandemic. **Methods:** In September 2022, we emailed an 8-question survey to Emerging Infections Network (EIN) physician members with infection prevention and hospital epidemiology responsibilities. The survey asked about the respondent’s primary hospital’s recommendations on transmission-based precautions, adjunctive measures to reduce MDRO transmission, and changes that occurred during the COVID-19 pandemic. We sent 2 reminder emails over a 1-month period. We used descriptive statistics to summarize the data and to compare results to a similar EIN survey (n = 336) administered in 2014 (Russell D, et al. doi:10.1017/ice.2015.246). **Results:** Of 708 EIN members, 283 (40%) responded to the survey, and 201 were involved in infection prevention. Most respondents were adult infectious diseases physicians (n = 228, 80%) with at least 15 years of experience (n = 174, 63%). Respondents were well distributed among community, academic, and nonuniversity teaching facilities (Table 1). Most respondents reported that their facility routinely used CP for methicillin-resistant *Staphylococcus aureus* (MRSA, 66%) and vancomycin-resistant *Enterococcus* (VRE, 69%), compared to 93% and 92% respectively, in the 2014 survey. Nearly all (>90%) reported using contact precautions for *Candida auris*, carbapenem-resistant Enterobacterales (CRE), and carbapenem-resistant *Acinetobacter* spp, but there was variability in the use of contact precautions for carbapenem-resistant *Pseudomonas aeruginosa* and extended-spectrum β-lactamase–producing gram-negative organisms. In 2014, 81% reported that their hospital performed active surveillance testing for MRSA, and in 2022 this rate fell to 54% (Table 2). The duration of contact precautions varied by MDRO (Table 3). Compared to 2014, in 2022 facilities were less likely to use contact precautions indefinitely for MRSA (18% vs 6%) and VRE (31% vs 11%). Also, 180 facilities (90%) performed chlorhexidine bathing in at least some inpatients and 106 facilities (53%) used ultraviolet light or hydrogen peroxide vapor disinfection at discharge in some rooms. Furthermore, 89 facilities (44%) reported institutional changes to contact precautions policies after the start of the COVID-19 pandemic that remain in place. **Conclusions:** Use of contact precautions for patients with MDROs is heterogenous, and policies vary based on the organism. Although most hospitals still routinely use contact precautions for MRSA and VRE, this practice has declined substantially since 2014. Changes in contact-precaution policies may have been influenced by the COVID-19 pandemic, and more specifically, contemporary public health guidance is needed to define who requires contact precautions and for what duration.

**Figure:**
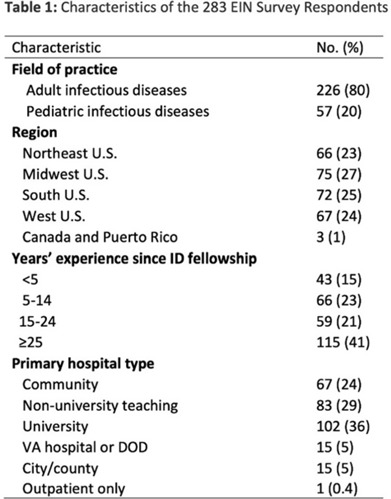

**Figure:**
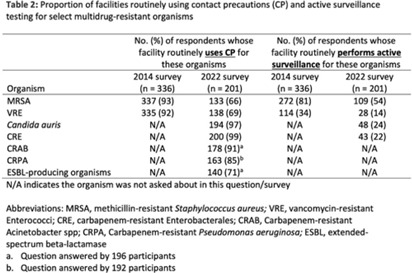

**Figure:**
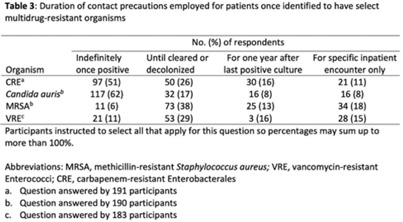

**Disclosures:** None

